# IL-4Rα Polymorphism Is Associated With Myasthenia Gravis in Chinese Han Population

**DOI:** 10.3389/fneur.2018.00529

**Published:** 2018-07-10

**Authors:** Ping Jiang, Yao-Xian Yue, Yu Hong, Yanchen Xie, Xiang Gao, Chuan-Kai Gu, Hong-Jun Hao, Yue Qin, Xiao-Jun Ding, Min Song, Hai-Feng Li, Xu Zhang

**Affiliations:** ^1^Department of Neurology, Affiliated Hospital of Qingdao University, Qingdao, China; ^2^Department of Neurology, Qilu Hospital of Shandong University, Jinan, China; ^3^Department of Clinical Medicine, University of Bergen, Bergen, Norway; ^4^Department of Neurology, Beijing Friendship Hospital, Capital Medical University, Beijing, China; ^5^ICU, Affiliated Hospital of Qingdao University, Qingdao, China; ^6^Department of Neurology, Peking University First Hospital, Beijing, China

**Keywords:** myasthenia gravis, interleukin-4 receptor, polymorphism, susceptibility, severity

## Abstract

Interleukin-4 (IL-4) is a potent growth and differentiation factor for B cells which play a vital role in the pathogenesis of myasthenia gravis (MG). IL-4 exerts its function by binding to three types of IL-4 receptor (IL-4R) complexes. IL-4Rα is the key component of the IL-4R complex. We hypothesize that polymorphism of IL-4Rα gene may be associated with the susceptibility and severity of MG. A Chinese cohort of 480 MG patients and 487 healthy controls were recruited. Polymorphisms of IL-4Rα gene were determined with SNPscan™ methods and compared between MG and control groups, as well as among MG subgroups. Rs2107356 and rs1805010 were found to be associated with adult thymoma associated MG, and rs1801275 was found to be associated with adult non-thymoma AChR-Ab positive MG. We did not found association between IL-4Rα polymorphism and the severity of MG. Genetic variations of IL-4Rα were found associated with the susceptibility of MG in Chinese Han population.

## Introduction

Myasthenia gravis (MG) is a T cell-dependent antibody-mediated autoimmune neuromuscular disorder and characterized by weakness and fatigability of skeletal muscles (1). Interleukin-4 (IL-4) is one of the Th2 cytokines which plays a vital role in the pathogenesis of MG. IL-4 is a potent growth and differentiation factor for B cells and stimulates class-switching and autoantibody production. IL-4 also takes part in the positive modulation of regulatory T cell (Treg) functions (2). IL-4 levels were found lower in MG patients compared with healthy controls (3). In mice genetically deficient in IL-4, long-lasting muscle weakness developed after a single immunization with acetylcholine receptor (AChR) (4).

IL-4 binds to three types of IL-4 receptor (IL-4R) complexes to exert its function. The type I IL-4R complex is present on lymphoid T cells, NK cells, basophils and mast cells; type II complex is found on non-lymphoid tumor cells, and type III complex is present on B cells and monocytes. Type I IL-4R is composed of two subunits, the IL-4Rα and the IL-2Rγc-common (γC) chain. Type II IL-4R is composed of two subunits, the IL-4Rα and the IL-13Rα1. In type III IL-4R, all three chains are present. Hence, IL-4Rα is the key component of all three complexes. When IL-4 binds to IL-4Rα, it recruits one of the other two chains in the complexes depending on the cell type and forms a heterodimer complex that initiates signal transduction (5). In experimental MG, IL-4R is upregulated in the myocytes following exposure to anti-AChR antibody, and demonstrated an increased responsiveness to IL-4, which implies the role of IL-4R in disease progression of experimental MG (6).

Till now, there was no evidence on the association of IL-4 gene polymorphism with MG (7). Polymorphisms of IL-4Rα were found to be associated with the susceptibility of autoimmune or inflammatory diseases such as rheumatoid arthritis (8–10), systemic lupus erythematosus (11), Graves' disease (12), eczema (13) and asthma (14–16), and with the severity and treatment effects of some of the diseases (9, 14). Pal et al. reported the frequency of rs1805010 GG genotype was significantly higher in MG patients than in healthy controls (17). The association between the polymorphism of IL-4Rα and MG remains to be confirmed independently. In the current study, we explore the potential association between polymorphism of IL-4Rα and the susceptibility and severity of MG in a Chinese cohort.

## Subjects and methods

### Study population

Four hundred and eighty MG patients and 487 healthy controls (HC) were recruited from Affiliated Hospital of Qingdao University and Beijing Friendship Hospital, Capital Medical University. All MG patients and healthy controls were Han Chinese population in origin and non-consanguineous. All patients met the diagnostic criteria of MG: the typical symptoms of fluctuating muscle weakness and positive result of neostigmine test as the essential conditions, and at least one of the supporting conditions: anti-AChR antibody (AChR-Ab) positive and/or the amplitude decrement of low frequency repetitive nerve stimulation (RNS) >10%. AChR-Ab was detected with ELISA kits (RSR Limited, Cardiff, UK). Patients were followed at least twice a year, with additional follow-ups on the worsening and within 2–3 months thereafter. Informed consent was obtained from the MG patients or their guardian and the controls. The study protocol was approved by ethical committees in the two hospitals.

**Figure 1:**
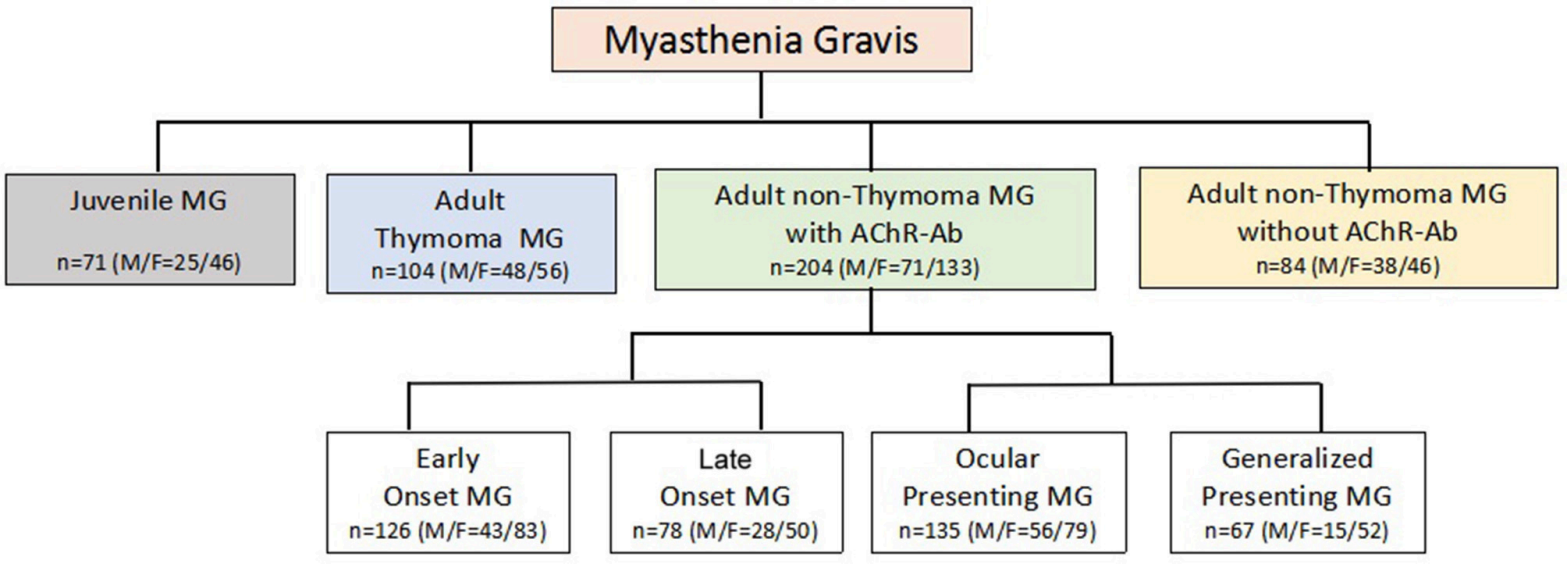
New Classification for MG subgroups.

MG patients were classified into subgroups according to essential clinical variables in MG: gender, onset age (<15/15–50/ > 50 years) (18), thymoma (CT and/or pathology identified), AChR-Ab (+/−), muscle involvement at onset (ocular/generalized), and severity at the maximal worsening (modified Oosterhuis score 0–2/3–5) (19) during the first 2 years after onset. Previous study suggested that 82% MG patients reached the maximum worsening within 2 years after onset (20), thus, we analyzed the maximum Oosterhuis scores only in patients with clinical duration of 2 years or more. Due to potential interaction among the essential clinical variables (21, 22), a new classification for subgroup (23) was also applied in analysis (Figure 1).

### SNPs selection and genotyping

Based on previous reports till July, 2013 and Pubmed SNP database, six SNPs (rs2107356, rs1805010, rs1805011, rs1805015, rs1801275, and rs8832) were selected in IL-4Rα gene. All SNPs met the requirement that the minor allele frequencies (MAF) > 5% (Table 1). Peripheral blood was collected in ethylenediamine tetraacetic acid (EDTA) vials, genomic DNA was isolated from peripheral blood using the Wizard genomic DNA purification kit (Biochain, Newark, CA, USA). SNP genotyping was performed with a custom-designed 2 × 48-Plex SNPscan™ Kit (Cat#: G0104, Genesky Biotechnologies Inc., Shanghai, China), which was based on double ligation and multiplex fluorescence PCR. Forty randomly selected samples from the patients and controls were typed for double-blinded quality control and were consistent with the original genotyping results.

**Table 1:** General characteristic of SNPs in IL-4R gene.

**Gene**	**SNPs**	**Alleles**	**Position(bp)**	**Function**	***P*HW**	**MAF^a^**
IL-4R	rs2107356	C > T	27312083	promoter	0.302	0.380
	rs1805010	G > A	27344882	missense	0.353	0.484
	rs1805011	A > C	27362551	missense	0.536	0.083
	rs1805015	T > C	27362859	missense	0.18	0.084
	rs1801275	A > G	27363079	missense	0.274	0.166
	rs8832	A > G	27364466	3′ UTR	0.194	0.486

a *MAF value of East Asian [EAS] in the 1000 Genomes Project (http://www.internationalgenome.org/)*.

### Statistical analysis

The online SNPstats software (http://bioinfo.iconcologia.net/snpstats/start.htm) was used to test the Hardy-Weinberg equilibrium (HWE) in HC. The χ^2^-test and Fisher exact test (SPSS 19.0) were used to compare allele frequencies and the SNPstats was used to compare genotype frequencies between MG and HC, between each MG subgroup and HC, between each pairs of MG subgroups (e.g., with thymoma vs. without thymoma), and among subgroups of the new classification and HC group. Bonferonni correction of the *p*-value was used for adjusting multiple tests. When there were significant differences in frequencies of alleles between MG subgroups and HC or among MG subgroups, logistic regression (SPSS 19.0) was used to adjust the potential confounders under codominant or additive models of inheritance. The criterion of significant difference was *P* < 0.05.

## Result

### General characteristics

Four hundred and eighty MG patients (291 females and 189 males) and 487 HC (238 females and 249 males) were enrolled in our study. The successful genotyping rates of six SNPs were 97.93 ~ 99.48%. The genotype distributions of six SNPs in HC were consistent with Hardy–Weinberg genetic equilibrium. The onset age of MG ranged 1 ~ 86 (median 40, interquartile range [IQR] 32). The age of HC group ranged 14 ~ 78 (median 45, IQR 24). 107 patients were with thymoma, 367 patients were without thymoma and 6 patients were not defined; 338 patients were AChR-Ab positive, 124 patients were AChR-Ab negative and 18 patients were not defined; 342 patients were ocular presenting at onset, 135 were generalized presenting and 3 patients were not defined. The disease duration of MG patients ranged 8–220 months (median 43, IQR 61). The mean number of follow-up each year was 6.5. The data of Oosterhuis score on the maximum worsening was available in 370 patients (76.9%). 216 patients were in mild subgroup (Oosterhuis score 0–2) and 154 patients were in severe subgroup (Oosterhuis score 3–5).

### Comparison between the whole MG group and healthy control group

Frequency of rs1801275 G allele was higher in MG than that in HC (*P* = 0.036, *OR* = 1.293, 95%CI 1.017–1.643, P_Bon_ = 0.216). There were significant differences in genotype frequencies of rs1801275 between MG and HC under codominant (*P* = 0.048) and additive (*P* = 0.015) models of inheritance (Table 2).

**Table 2:** Distribution of SNPs among all 480 MG patients without any subgroup distinction and 487 controls.

**SNPs**	**MAF**		**Genotypes**	**Genotype frequencies**		***P*****-value**	

	**MG**	**HC**		**MG**	**HC**	**Codominant**	**Additive**
rs2107356	0.38	0.37	CC	174 (37.6)	200 (41.3)	0.46	0.43
			CT	222 (48.0)	214 (44.2)		
			TT	67 (14.5)	70 (14.5)		
rs1805010	0.5	0.48	GG	127 (26.7)	135 (27.7)	0.71	0.42
			GA	222 (46.7)	233 (47.8)		
			AA	126 (26.5)	119 (24.4)		
rs1805011	0.07	0.08	AA	409 (86.3)	410 (84.2)	0.51	0.52
			CA	62 (13.1)	75 (15.4)		
			CC	3 (0.6)	2 (0.4)		
rs1805015	0.07	0.08	TT	409 (86.3)	409 (84.0)	0.26	0.49
			CT	62 (13.1)	77 (15.8)		
			CC	3 (0.6)	1 (0.2)		
rs1801275	0.19	0.15^a^	AA	305 (64.8)	348 (71.5)	0.048	0.015
			GA	156 (33.1)	131 (26.9)		
			GG	10 (2.1)	8 (1.6)		
rs8832	0.45	0.42	AA	146 (30.7)	157 (32.4)	0.24	0.18
			GA	233 (49.0)	250 (51.5)		
			GG	96 (20.2)	78 (16.1)		

a *MG vs. HC, P = 0.036, OR = 1.293 95%CI (1.017–1.643), P_Bon_ = 0.216*.

### Comparison between MG subgroups and healthy control group and among MG subgroups

Frequencies of rs2107356 T allele and rs1805010 A allele were significantly higher in thymoma (+) subgroup than those in HC and in thymoma (−) subgroup both after Bonferonni correction. There were significant differences in genotype frequencies of rs2107356 and rs1805010 between thymoma (+) subgroup and HC and between thymoma (+) and thymoma (−) subgroups under codominant and additives models. There were no significant differences in frequencies of rs2107356 T allele and rs1805010 A allele between thymoma (−) subgroup and HC (Table 3).

**Table 3:** Frequencies of alleles and genotypes of rs1805010, rs1801275 and rs2107356 in MG subgroups and controls [case (%)].

	**Control**	**Male**		**Female**		**Anti-AchR Ab**		**Thymoma**	

		**MG**	**control**	**MG**	**control**	**Positive**	**Negative**	**positive**	**negative**
Number	487	189	249	291	238	338	124	107	367
***Rs2107356***	484^*^	***180***	***247***	***283***	***237***	***325***	***120***	***102***	***355***
CC	200(41.3)	66(36.7)	105(42.5)	108(38.2)	95(40.1)	122(37.5)	44(36.7)	31(30.4)	141(39.7)
CT	214(44.2)	88(48.9)	109(44.1)	134(47.4)	105(44.3)	154(47.4)	59(49.2)	45(44.1)	174(49.0)
TT	70(14.5)	26(14.4)	33(13.4)	41(14.5)	37(15.6)	49(15.1)	17(14.2)	26(25.5)	40(11.3)
T allele	0.37	0.39	0.35	0.38	0.38	0.39	0.39	0.48^a^	0.36^b^
***Rs1805010***	***487***	***186***	***249***	***289***	***238***	***334***	***124***	***106***	***363***
GG	135(27.7)	43(23.1)	64(25.7)	84(29.1)	71(29.8)	88(26.4)	30(24.2)	20(18.9)	105(28.9)
GA	233(47.8)	91(48.9)	125(50.2)	131(45.3)	108(45.4)	161(48.2)	53(42.7)	45(42.5)	175(48.2)
AA	119(24.4)	52(28.0)	60(24.1)	74(25.6)	59(24.8)	85(25.4)	41(33.1)	41(38.7)	83(22.9)
A allele	0.48	0.52	0.49	0.48	0.47	0.5	0.54	0.6^c^	0.47^d^
***Rs1801275***	***487***	***183***	***249***	***288***	***238***	***331***	***122***	***104***	***361***
AA	348(71.5)	108(59.0)	175(70.3)	197(68.4)	173(72.7)	202(61.0)	87(71.3)	66(63.5)	236(65.4)
AG	131(26.9)	68(37.2)	69(27.7)	88(30.6)	62(26.1)	120(36.3)	34(27.9)	36(34.6)	117(32.4)
GG	8(1.6)	7(3.8)	5(2.0)	3(1.0)	3(1.3)	9(2.7)	1(0.8)	2(1.9)	8(2.2)
G allele	0.15	0.22	0.16	0.16	0.14	0.21^e^	0.15	0.19	0.18
	**Control**		**Onset age (year)**			**Onset involvement**		**Oosterhuis score**	
			<**15**	**15–50**	>**50**	**Ocular**	**Generalized**	**0–2**	**3–5**
Number	487		71	253	156	342	135	216	154
***Rs2107356***	***484***		***68***	***247***	***148***	***330***	***130***	***209***	***149***
CC	200(41.3)		29(42.6)	93(37.7)	52(35.1)	126(38.2)	47(36.1)	81(38.8)	62(40.3)
CT	214(44.2)		32(47.1)	113(45.7)	77(52.0)	160(48.5)	60(46.1)	103(49.3)	65(43.6)
TT	70(14.5)		7(10.3)	41(16.6)	19(12.8)	44(13.3)	23(17.7)	25(12.0)	24(16.1)
T allele	0.37		0.34	0.39	0.39	0.38	0.41	0.37	0.38
***Rs1805010***	***487***		***70***	***252***	***153***	***337***	***135***	***216***	***152***
GG	135(27.7)		21(30.0)	69(27.4)	37(24.2)	88(26.1)	39(28.9)	56(25.9)	45(29.6)
GA	233(47.8)		27(38.6)	109(43.2)	86(56.2)	161(48.4)	57(42.2)	100(46.3)	74(48.7)
AA	119(24.4)		22(31.4)	74(29.4)	30(19.6)	86(25.5)	39(28.9)	60(27.8)	33(21.7)
A allele	0.48		0.51	0.51	0.48	0.5	0.5	0.51	0.46
***Rs1801275***	***487***		***69***	***252***	***150***	***337***	***131***	***214***	***152***
AA	348(71.5)		51(73.9)	163(64.7)	91(60.7)	216(64.1)	88(67.2)	141(65.9)	98(65.1)
AG	131(26.9)		17(24.6)	83(32.9)	56(37.3)	114(33.8)	40(30.5)	70(32.7)	49(32.2)
GG	8(1.6)		1(1.4)	6(2.4)	3(2.0)	7(2.1)	3(2.3)	3(1.4)	5(3.3)
G allele	0.15		0.14	0.19	0.21	0.19	0.18	0.18	0.19

* *Italic bold refers to the number of successfully genotyped patients with adequate clinical data in each group*.

a . Thymoma (+) MG vs. HC, P = 0.003, OR = 1.572 (1.160–2.132), P_Bon_ = 0.018 codominant model, thymoma (+) MG vs. HC, P = 0.017 additive model, thymoma (+) MG vs. HC, P = 0.0055

b . Thymoma (+) MG vs. Thymoma (−) MG, P = 0.002, OR = 1.627 (1.188–2.229), P_Bon_ = 0.012 codominant model, thymoma (+) MG vs. thymoma (−) MG, P = 0.0025 additive model, thymoma (+) MG vs. thymoma (−) MG, P = 0.0023

c . Thymoma (+) MG vs. HC, P = 0.002, OR = 1.596 (1.180-2.158), P_Bon_ = 0.012 codominant model, thymoma (+) MG vs. HC, P = 0.008 additive model, thymoma (+) MG vs. HC, P = 0.003

d . Thymoma (+) MG vs. Thymoma (−) MG, P = 0.001, OR = 1.687 (1.236–2.302), P_Bon_ = 0.006 codominant model, thymoma (+) MG vs. thymoma (−) MG, P = 0.004 additive model, thymoma (+) MG vs. thymoma (−) MG, P = 0.0012

e . AChR-Ab (+) MG vs. HC, P = 0.003, OR = 1.482 (1.146–1.915), P_Bon_ = 0.018 codominant model, AChR Ab (+) MG vs. HC, P = 0.0032 *additive model, AChR Ab (+) MG vs. HC, P = 0.0007*.

Frequency of rs1801275 G allele was significantly higher in AChR-Ab (+) subgroup than that in HC after Bonferonni correction. There were significant differences in genotype frequencies of rs1801275 between AChR-Ab (+) subgroup and HC under codominant and additive models of inheritance. Frequency of rs1801275 G allele was significantly higher in male MG patients than that in male controls (*P* = 0.017, *OR* = 1.531, 95%CI 1.086–2.160), and higher in AChR-Ab (+) subgroup than that in AChR-Ab (−) subgroup (*P* = 0.039, *OR* = 1.522, 95%CI 1.020–2.271). After Bonferonni correction, the differences were not significant. There were no significant differences in allele and genotype frequencies between AChR-Ab (−) subgroup and HC (Table 3).

### Adjustment of potential confounders in clinical variable based subgroup analysis

Logistic regression analysis was performed with thymoma (±) as dependent variables, and with onset age (<15/15–50/ > 50 years, <15 as reference), gender (male/female), AChR-Ab (±), muscle involvement at onset (ocular/generalized) and genotypes of rs2107356 or rs1805010 separately (under codominant or additives model) as independent variables. Onset age, AChR-Ab (+) and genotypes of rs2107356 and rs1805010 were found as independent risk factors for the presence of thymoma (Tables 4).

**Table 4:** Logistic regression analysis in subgroups classified by thymoma.^a^

**Variables**	**Regression coefficient**	**Standard error**	**Wald *x*^2^-value**	***P*-value**	**OR**	**95%CI**
Onset age (< 15 as reference)			10.448	0.005		
15–50 year	1.964	0.621	10.017	0.002	7.128	2.112–24.056
>50 year	1.682	0.636	6.991	0.008	5.375	1.545–18.699
AChR-Ab positive	1.146	0.331	11.994	0.001	3.145	1.644–6.015
rs1805010	0.535	0.168	10.198	0.001	1.708	1.230–2.372
Onset age (< 15 as reference)			10.588	0.005		
15–50 year	1.846	0.620	8.863	0.003	6.335	1.879–21.358
>50 year	1.392	0.638	4.764	0.029	4.028	1.153–14.077
AChR-Ab positive	1.024	0.331	9.596	0.002	2.785	1.457–5.323
rs2107356	0.440	0.172	6.498	0.011	1.552	1.107–2.176

a *The results were same under both codominant model and additive model*.

Logistic regression analysis was performed with AChR-Ab (±) as dependent variables, and with onset age, gender, thymoma, muscle involvement at onset and genotypes (under codominant or additive model) as independent variables. Thymoma, ocular presenting and rs1801275 genotypes were found as independent risk factors for AChR-Ab positivity (Table 5).

**Table 5:** Logistic regression analysis in subgroups classified by AChR-Ab.^a^

**Variables**	**Regression coefficient**	**Standard error**	**Wald *x*^2^-value**	***P*-value**	**OR**	**95%CI**
Onset age (< 15 as reference)			1.329	0.515		
15–50	0.358	0.314	1.304	0.254	1.431	0.774–2.645
>50	0.303	0.334	0.827	0.363	1.354	0.704–2.604
Thymoma	1.054	0.329	10.283	0.001	2.871	1.507–5.469
Ocular presenting	0.693	0.286	5.881	0.015	0.500	0.286–0.876
rs1801275	0.515	0.225	5.246	0.022	1.674	1.077–2.600

a *The results were same under both codominant model and additive model*.

### Comparison between MG subgroups and healthy control group and among MG subgroups in the new classification

The new classification (Figure 1) used the combination of essential clinical features of MG to sort biological and clinical meaningful subgroups. It ensured any MG patients with sufficient data to be allocated into one subgroup and only in one subgroup. According to the new classification, 71 patients were juvenile onset MG (JMG). In 409 adult onset MG patients, 104 were with thymoma. In 300 adult MG patients without thymoma, 84 were AChR-Ab negative. In 204 non-thymoma AChR-Ab positive patients, 126 patients were early onset MG (EOMG, onset age 15–50) and 78 were late onset MG (LOMG, onset age > 50); 135 patients were ocular presenting and 67 were generalized presenting. The other patients had not relevant information to be defined into subgroups in this new classification.

Frequencies of rs2107356 T allele and rs1805010 A allele in adult thymoma subgroup was significantly higher than those in HC and in adult non-thymoma subgroup after Bonferonni correction. There were significant differences in genotype frequencies of rs2107356 and rs1805010 between adult thymoma subgroup and HC and between adult thymoma subgroup and adult non-thymoma subgroup under codominant and additive models (*p* < 0.05, Table 6). There was no difference in allele and genotype frequencies of the two SNPs between adult non-thymoma subgroup and HC.

**Table 6:** Frequencies of alleles and genotypes of rs1805010, rs1801275, and rs2107356 in MG subgroups of a new classification and the control group [case (%)].

	**HC**	**Juvenile (< 15)**	**Adult (≥15)**								
				**Thymoma**	**Non-thymoma**						
						**AchRAb (−)**	**AchRAb (+)**				
								**15–50**	**>50**	**Ocular**	**Generalized**
Number	487	71	409	104	300	84	204	126	78	135	67
***rs2107356***	484^*^	***68***	***395***	***99***	***291***	***81***	***198***	***123***	***75***	***133***	***63***
CC	200 (41.3)	29 (42.6)	145 (36.7)	30 (30.3)	114 (39.2)	29 (35.8)	79 (39.9)	52 (42.3)	27 (36.0)	57 (42.9)	19 (30.2)
CT	214 (44.2)	32 (47.1)	190 (48.1)	43 (43.4)	144 (49.5)	40 (49.5)	98 (49.5)	58 (47.2)	40 (53.3)	58 (43.6)	37 (58.7)
TT	70 (14.5)	7 (10.3)	60 (15.2)	26 (26.3)	33 (11.3)	12 (14.8)	21(10.6)	13 (10.6)	8 (10.7)	18 (13.5)	7 (11.1)
T allele	0.37	0.34	0.39	0.48^a^	0.36^b^	0.40	0.35	0.34	0.37	0.35	0.40
***rs1805010***	***487***	***70***	***405***	***103***	***297***	***84***	***201***	***126***	***75***	***134***	***66***
GG	135 (27.7)	21 (30.0)	106 (26.2)	20 (19.4)	85 (28.6)	18 (21.4)	60 (29.9)	42 (33.3)	18 (24.0)	38 (28.4)	13 (19.7)
GA	233 (47.8)	27 (38.6)	195 (48.1)	43 (41.7)	150 (50.5)	42 (50.0)	103 (51.2)	58 (46.0)	45 (60.0)	65 (48.5)	35 (53.0)
AA	119 (24.4)	22 (31.4)	104 (25.7)	40 (38.8)	62 (20.9)	24 (28.6)	38 (18.9)	26 (20.6)	12 (16.0)	1 (23.1)	18 (27.3)
A allele	0.48	0.51	0.50	0.60^c^	0.46^d^	0.54	0.45	0.44	0.46	0.47	0.43
***rs1801275***	***487***	***69***	***402***	***101***	***296***	***83***	***201***	***126***	***75***	***133***	***66***
AA	348 (71.5)	51 (73.9)	254 (63.2)	63 (62.4)	189 (63.9)	57 (68.7)	122 (60.7)	77 (61.1)	45 (60.0)	86 (64.7)	39 (59.1)
GA	131 (26.9)	17 (24.6)	139 (34.6)	36(35.6)	100(33.8)	25(30.1)	73 (36.3)	45 (35.7)	28 (37.3)	47 (35.3)	24 (36.4)
GG	8 (1.6)	1 (1.4)	9 (2.2)	2(2.0)	7(2.4)	1 (1.2)	6 (3.0)	4 (3.2)	2 (2.7)	0 (0)	3 (4.5)
G allele	0.15	0.14	0.20	0.20	0.19	0.17	0.21^e^	0.21	0.21	0.18	0.23

* *Italic bold refers to the number of successfully genotyped patients with adequate clinical data in each group*.

a . adult thymoma MG vs. HC, P = 0.003, OR = 1.600 (1.176-2.177), P_Bon_ = 0.018 codominant model, adult thymoma MG vs. HC, P = 0.0056 additive model, adult thymoma MG vs. HC, P = 0.0017

b . adult thymoma MG vs. adult non-thymoma MG, P = 0.003, OR = 1.634 (1.179–2.264), P_Bon_ = 0.018 codominant model, adult thymoma MG vs. non-thymoma MG, P = 0.0025 additive model, adult thymoma MG vs. non-thymoma MG, P = 0.0032

c . *adult thymoma MG vs. HC, P = 0.003, OR = 1.583 (1.166–2.148), P_Bon_ = 0.018* codominant model, adult thymoma MG vs. HC, P = 0.0034 additive model, adult thymoma MG vs. HC, P = 0.0013

d . *adult thymoma MG vs. adult non-thymoma MG, P = 0.001, OR = 1.731 (1.255–2.388), P_Bon_ = 0.006* codominant model, adult thymoma MG vs. non-thymoma MG, P = 0.0025 additive model, adult thymoma MG vs. adult non-thymoma MG, P = 0.0012

e . *adult non-thymoma AChR-Ab positive MG vs. HC, P = 0.006, OR = 1.509 (1.121-2.030), P_Bon_ = 0.036* codominant model, adult non-thymoma AChR-Ab positive MG vs. HC, P = 0.011 *additive model, adult non-thymoma AChR-Ab positive MG vs. HC, P = 0.0026*.

Frequency of rs1801275 G allele was significantly higher in adult non-thymoma AChR-Ab positive subgroup than that in HC after Bonferonni correction. There were significant differences in genotype frequencies of rs1801275 between adult non-thymoma AChR-Ab positive subgroup and HC group under codominant and additive models (Table 6). Frequency of rs1801275 G allele was significantly higher in adult non-thymoma AChR-Ab positive MG than in adult non-thymoma AChR-Ab negative MG (*P* = 0.023). After Bonferonni correction, the difference was not significant. There were no significantly difference in rs1801275 G allele and genotype frequencies between adult non-thymoma AChR-Ab negative subgroup and HC group. Frequency of rs1801275 G allele was higher in EOMG, LOMG, ocular presenting and generalized presenting subgroups than those in HC, but there were no allelic differences between EOMG subgroup and LOMG subgroup, and between ocular presenting subgroup and generalized presenting subgroup (Table 6).

## Discussion

The current study tried to explore the association between candidate SNPs and the susceptibility and severity of MG in a representing MG cohort. Significant association was found between rs2107356 and rs1805010 and thymoma subgroup, and between rs1801275 and AChR-Ab positivie subgroup. No association was found among these SNPs and severity of MG. In previous subgroup analysis (21, 22), we found any single clinical variable of MG may be confound with other clinical variables, therefore, we adjusted these variables to analysis whether genotypes are independent risk factors for the association with a specific clinical feature. We found the association of rs2107356 and rs1805010 with thymoma and association of rs1801275 with AChR-Ab positivity. We then used the new classification which was based on the current insight of pathogenic mechanism and subgroup reasoning of MG to minimize the interaction among different clinical variables. We found association of rs2107356 and rs1805010 with adult thymoma associated MG; and found association of rs1801275 with adult non-thymoma AChR-Ab positivie MG.

Although allele and genotype frequencies of rs2107356 and rs1805010 were significantly higher in the adult thymoma MG subgroup than those in HC and in adult non-thymoma MG subgroup, there was no difference between adult non-thymoma MG subgroup and HC. Thymoma is a tumor of thymic epithelium. The association of thymoma and MG is well-known (24). As far as we known, this is the first time to find the association of these two SNPs with thymoma.

Rs2107356 is a promoter SNP which might increase the expression of IL-4R, which should be studied in the future. Rs2107356 was also found associated with gastric cancer (25) and multiple myeloma (26). Rs1805010 is a gain-of-function mutation in the extracellular domain of IL-4Rα subunit. This variant enhances IL-4 signaling by keeping STAT6 transcription factor abnormally active, which was thought not due to modulation of intracellular mechanisms (27). Rs1805010 was also found associated with renal cell cancer (28) and cervical cancer (29). Most of the association was reported in epithelic tumors. IL-4 supports cellular proliferation of human tumor cell lines in a concentration-dependent manner. IL-4R blockade dramatically reduces lymphatic and hematogenous metastases of some kinds of tumors *in vivo*. IL-4 also acts as a survival factor on tumor cells by increasing the expression of anti-apoptotic molecules. These effects of IL-4 on tumor cells predominantly signals through the type II IL-4R complex (5). The role of IL-4R on the pathogenesis in thymoma might resemble with those in other tumors.

In our study, frequency of G allele in rs1801275 was higher in MG patients than that in HC group, especially in adult non-thymoma AChR-Ab positive MG subgroup. Genotypes of rs1801275 were found to be independent factors for the presence of AChR-Ab in MG. The rs1801275 is another gain-of-function mutation in the intracellular domain of IL-4Rα, which enhancs IL-4Rα signal transduction via STAT6 pathway, which has important effects on immune modulating (30, 31). This variant was also found associated with IL-4-directed and IL-6-dependent subversion of Treg cells into Th17-like cells (16). Th17 cell is critical in the pathogenesis of MG (32, 33), The effect of rs1801275 on the balance between Treg and Th17 may explain our results on the association of this mutant with AChR-Ab positive MG.

Our study had limitations. We did not enroll thymoma patients without MG, therefore, the association between IL-4Rα gene polymorphism and thymoma should be further confirmed. Moreover, previous studies have shown that the T cell receptors (TCRs) of MG with thymoma are different from those of MG without thymoma (34). Whether IL-4Rα plays a role in the growth of thymoma and induces the T cells expressing specific TCRs to metastasize to periphery to participate in the production of AChR-Ab wait for further researches.

In conclusion, in Chinese Han population, rs2107356 and rs1805010 were found to be associated with adult thymoma associated MG, and rs1801275 was found to be associated with adult non-thymoma AChR-Ab positive MG.

## Ethics statement

This study was carried out in accordance with the recommendations of ethical committees of Affiliated Hospital of Qingdao University, and Beijing Friendship Hospital, Capital Medical University. The protocol was approved by the ethical committees of Affiliated Hospital of Qingdao University, and Beijing Friendship Hospital, Capital Medical University. All subjects gave written informed consent in accordance with the Declaration of Helsinki.

## Author contributions

PJ, Y-XY, and H-FL conceptualized and designed the study, interpreted the data, and wrote the manuscript. Y-XY and YH designed the genotyping experiment. H-JH performed the AChR antibody testing. PJ, Y-XY, and YH performed statistical analysis. YQ, X-JD, and MS contributed to discussion. H-FL, YX, XG, XZ, and C-KG diagnosed and treated patients in this study. YX, XG, and Y-XY maintained the database.

### Conflict of interest statement

The authors declare that the research was conducted in the absence of any commercial or financial relationships that could be construed as a potential conflict of interest.
